# Behavioural and electroencephalographic assessment of captive-bolt stunning in kangaroo pouch young

**DOI:** 10.1017/awf.2026.10070

**Published:** 2026-03-26

**Authors:** Trudy M Sharp, Steven R McLeod, Troy J Gibson

**Affiliations:** 1 Vertebrate Pest Research Unit, New South Wales Department of Primary Industries and Regional Development, Australia; 2 Animal Welfare Science and Ethics Group, The Royal Veterinary College Department of Pathobiology and Population Sciences, United Kingdom

**Keywords:** Animal welfare, blunt force trauma, captive bolt, culling, euthanasia, humaneness, kangaroo harvesting, pouch young

## Abstract

During commercial harvesting (shooting) of kangaroos, pouch young of shot females must be euthanased to prevent suffering. The current euthanasia method, manually applied concussive (or blunt force) trauma to the head, can be effective but is not always applied consistently and is often perceived by observers to be inhumane. The captive-bolt device (CBD), which fires a steel bolt that either penetrates or impacts the skull, could provide a more suitable alternative. We reviewed a range of potentially suitable CBDs and assessed the effectiveness of four types on live animals. Effectiveness of CBDs was determined by assessing behaviour, electroencephalogram (EEG) and evaluating brain and skull trauma post mortem. Pouch young were also euthanased using manual blunt force trauma for comparison. Shooting with a *penetrating* CBD produced brain activity that was inconsistent with consciousness in 100% (n = 20) of animals. Behavioural indicators of consciousness and normal-like EEG were not detected after shooting with the CBD and damage to the brain was extensive. Seven out of 29 (24%) joeys shot with a *non-penetrating* CBD were either still breathing (n = 1) or recovered breathing (n = 6) after shooting. All seven animals had no or only mild damage to the medulla. We conclude that a cartridge-powered, penetrating CBD and manual blunt force trauma can both achieve immediate unconsciousness in pouch young, but a second step to exsanguinate the animal must still be performed. Penetrating CBDs are preferred to manual blunt force trauma since they are more repeatable, less reliant upon operator skill and confidence and more likely to reduce animal (and observer) distress.

## Introduction

In Australia, views of kangaroos are diverse. They are culturally and spiritually significant to Aboriginal people, an important national icon that should be preserved, but also a pest that needs to be managed and a valuable resource that can be used as a source of meat and other products (Thomsen *et al.* 2006; McLeod & Hacker 2020). Since the early 19^th^ century kangaroos have been culled, often when they have become overabundant, to control damage to pastoral lands. In the early 1970s, a regulated commercial kangaroo industry was developed which allowed for the sustainable culling by shooting — or ‘harvesting’ as it is commonly called — of wild, free-ranging kangaroos (and in Tasmania, some species of wallaby) to gain an economic return from the sale of their meat and/or skins. Harvesting of kangaroos thus serves a dual purpose, to provide a management option for landholders wanting to reduce pastoral damage and to make use of a sustainable resource (Anon 2020). The number of kangaroos that can be taken for commercial purposes is set by annual harvest quotas based on kangaroo population monitoring. In some situations, mostly for damage mitigation, kangaroos are also killed by shooting for non-commercial purposes, and the carcases or skin are not sold (Commonwealth of Australia 2008). Regardless of the reason for killing, all kangaroos and wallabies are protected native fauna and can only be shot under an appropriate licence or permit issued by a state or territory government.

The kangaroo species that are harvested in mainland Australia are: red kangaroo (*Osphranter rufus*) in Queensland (Qld), New South Wales (NSW), South Australia (SA) and Western Australia (WA); eastern grey kangaroo (*Macropus giganteus*) in Qld, NSW, and Victoria (Vic); western grey kangaroo (*M. fuliginosus*) in NSW, SA, Vic and WA; common wallaroo or euro (*M. robustus*) in Qld, NSW and SA. Wallaby species harvested in Tasmania are Bennett’s wallaby (a sub species of red-necked wallaby) (*Notomacropus rufogriseus rufogriseus*), Tammar wallaby (*Notomacropus eugenii*) and Tasmanian pademelon (or rufous-bellied pademelon) (*Thylogale billardierii*). Products derived from kangaroos are used domestically and overseas. Best practice for commercial harvesting of kangaroos is outlined in a National Code of Practice for the Humane Shooting of Kangaroos and Wallabies for Commercial Purposes (Anon 2020), herein referred to as the Code. The Code contains standards that must be achieved to minimise animal pain and suffering during the harvesting process. It provides specifications for firearms and ammunition and guidelines on procedures for shooting target kangaroos and euthanasia of injured animals, pouch young and young-at-foot (Sharp & McLeod 2020). All commercial kangaroo harvesters (herein referred to as kangaroo shooters) must comply with the Code as a condition of their licence or permit to harvest issued by state and territory regulators.

The Code stipulates that dependent young must be euthanased when their mother is killed. Due to the size of the commercial harvest, the potential number of young that must be euthanased is large. For example, considering only the commercial harvest of kangaroos in 2023 for Queensland, NSW, and South Australia, of the 1,314,702 kangaroos harvested, approximately 213,800 were female (Department of Climate Change, Energy, the Environment and Water 2024; Department for Environment and Water 2024; Queensland Parks and Wildlife Service 2024). Even if only 50% of females are carrying a pouch young (and this is most likely an underestimate of the percentage with pouch young), then more than 100,000 pouch young would have been euthanased in those three states in 2023. Therefore, there is a strong incentive to reduce, as much as possible, the welfare impacts on dependent young by using a method of euthanasia that reduces suffering.

Acceptable euthanasia methods currently prescribed in the Code are decapitation or cervical dislocation for pouch young that have no fur and closed eyes; and manually applied concussive blow (also called blunt force trauma) to the head for partially furred to fully furred pouch young (Anon 2020). Different euthanasia methods are used for different stages of development because there is evidence to suggest that unfurred pouch young that still have closed eyelids are not yet able to consciously perceive pain due to the immaturity of their neurological system, whereas young that are starting to develop fur and open their eyes can experience a range of different sensations, including physical pain (Diesch *et al.* 2008). In *sentient* animals (i.e. those that can perceive sensations such as pain), decapitation and dislocation are likely to cause a period of suffering before death, therefore a concussive blow to the head is currently used to rapidly cause irreversible unconsciousness and death. Observations of kangaroo shooters using manual blunt force trauma to euthanase pouch young have shown that, although joeys will experience a short period of anxiety and fear caused by the need to remove them from the pouch, the method is sufficiently effective in causing a rapid death when applied correctly, although there are some areas where improvements need to be made (i.e. by standardising techniques to reduce operator variability) (Sharp 2015). Significant disadvantages of the blunt force trauma method are that it is visually unpleasant, operators can be uncomfortable carrying it out and it is often perceived to be brutal, therefore it is often criticised (Dalla Costa *et al.* 2019). An alternative to manually applied blunt force trauma is the captive-bolt device (CBD) which is a more reliable and repeatable euthanasia method (Dalla Costa *et al.* 2020). In addition, although CBDs have a similar mode of action to manual blunt force trauma, the mode of action of non-penetrating CBDs is mechanical blunt force trauma, their use is likely to be perceived as a more ‘clinical’ approach to euthanasia.

The application of a CBD is a mechanical stunning (and sometimes also killing) method used to render an animal immediately unconscious by producing a severe blow to the skull. Cartridges with either a nitrocellulose propellant, compressed air, propane gas or a spring under tension are used to drive the bolt against or through the skull (Lambooij & Algers 2016). CBDs are commonly used in slaughterhouses on farmed animals including cattle, sheep, goats, pigs, horses, turkeys, rabbits, etc and also in other situations and with other species where euthanasia or depopulation of animals is required (American Veterinary Medical Association [AVMA] 2020; Baier & Wilson 2020). There are two types of CBDs: *penetrating* captive bolts that fire a cylindrical metal rod through the skull and into the brain; and *non-penetrating* bolts that have a wide, mushroom-shaped head that strikes the skull but does not enter the brain. In general, a penetrating CBD is recommended for most species because it is more reliable at delivering an effective stun and the bolt entering and exiting the brain causes extensive trauma and disruption to brain tissue, making recovery unlikely (Baier & Wilson 2020). Non-penetrating CBDs, which deliver a mechanical blunt force trauma, often only stun animals and therefore are generally not effective as a sole means of euthanasia, however they can be effective at killing smaller animals such as poultry (Gibson *et al.* 2018; Woolcott *et al.* 2018; Bandara *et al.* 2019), neonatal goat kids (Sutherland *et al.* 2016; Grist *et al.* 2018c), lambs (Grist *et al.* 2018a) and piglets (Grist *et al.* 2017, 2018b) without having to use a secondary method such as exsanguination (AVMA 2020). The effectiveness of different types of CBDs should therefore be evaluated on the species and age group it is intended to be used.

CBDs are considered much safer to use compared with rifles or pistols since a retractable bolt is used to stun and cause damage to the brain rather than a free projectile. However, a disadvantage of CBDs over firearms is that the animal needs to be closely approached, restrained and the head held still to allow for proper placement of the shot and to prevent operator injury (Jubb 2013). Selection of the most appropriate CBD must consider the energy delivered by the bolt. It is the kinetic energy that is imparted to the cranium by the bolt that produces unconsciousness, while the actual physical damage by the bolt to specific brain structures is responsible for producing irrecoverable unconsciousness (Gibson *et al.* 2015b). In addition to the performance characteristics of the CBD, effective captive-bolt stunning is dependent upon the accurate placement of the shot as well as operator skill and experience. Correct anatomical placement of the device to cause sufficient disruption to the brainstem and good marksmanship have been found to be definitive factors in the effective and humane use of CBDs in a range of species (e.g. see Gibson *et al.* 2012; Sutherland *et al.* 2016). Optimal anatomical placement of the bolt can vary between species and depend on the device used; therefore, we reinforce that it is essential that different CBDs are evaluated on the species and age group upon which they are intended to be applied.

To our knowledge, there have been two previous studies to investigate the use of a CBD for the stunning of kangaroo young. Sharp *et al.* (2015) tested a spring-operated CBD on the heads of pouch young cadavers and 21 live pouch young, but this device was not found to be acceptable. In addition, Hampton (2019) tested a penetrating, explosive-powered CBD (CASH® Special 0.22) on 28 furred pouch young and reported that 27 of these were rendered immediately insensible. The spring-operated device tested by Sharp *et al.* (2015) had poor effectiveness at inducing irrecoverable unconsciousness and was found to deliver much less kinetic energy when compared to cartridge-powered devices. The study by Hampton (2019) did not report times to death or if the CBD was sufficient to cause death or a secondary method needed to be applied. Also, Hampton did not calculate confidence or credible intervals for the reported mean stunning rate, reporting only a point estimate that 96% (27/28) of pouch young were effectively stunned. We calculated the 95% credible interval for the study and found that the lower credible interval was only 0.94. Given that 95% is the minimum acceptable threshold for achieving insensibility with one shot using captive-bolt devices in domestic animal abattoirs (Grandin 2010), the efficacy results from this study were too low for CBDs to be recommended and more data were therefore required (Sharp & McLeod 2020).

Effectiveness of CBDs at inducing unconsciousness and death can be determined by observing animal behaviour (e.g. breathing, reflexes, convulsions, leg paddling etc) and using electroencephalography/electroencephalogram (EEG) both before and after stunning. Following stunning, several observable indicators allow assessment of the state of consciousness or unconsciousness of the mammalian animal. These are indirectly associated with structures of the brain that are involved with maintaining consciousness, namely the brainstem (which includes the midbrain, pons and medulla) and/or the cerebral cortex (Terlouw 2020). According to Terlouw *et al.* (2016), animals are definitely conscious when they exhibit *any* of these six indicators: standing posture; head or body righting reflex; voluntary vocalisation; spontaneous blinking (no touching); eye pursuit; and response to threat or menace test (no touching). Animals that are unconscious and brain-dead will not have: a corneal reflex; eyelash reflex (in response to touch); or rhythmic breathing. To be certain that an animal is unconscious and therefore *insensible* or unable to perceive pain, indicators of consciousness must be absent, and indicators of unconsciousness must be present.

When testing for signs of sensibility in alpacas (*Vicugna pacos*) that had been shot with a penetrating captive bolt, Gibson *et al.* (2015c) checked for the presence or absence of immediate collapse; righting reflex; rhythmic breathing; corneal reflex; palpebral reflex; eyeball rotation; nystagmus; jaw muscle tension; convulsions; leg kicking; and cardiac arrest (by palpation of the chest). Animals were classified as sensible immediately after shooting if either rhythmic breathing or failure to collapse was present and/or if two of more of the following parameters were present: positive corneal reflex, positive palpebral reflex, eyeball rotation and nystagmus. They defined shallow depth of concussion as the presence of eyeball rotation, nystagmus or an intermittently positive corneal or palpebral reflex (Gibson *et al.* 2015c).

Over the past four decades, studies to evaluate stunning and slaughter methods have used EEG to record the electrical activity of the brain (e.g. Blackmore & Newhook 1982; Blackmore *et al.* 1995; Gibson *et al.* 2009b, 2019; Sutherland *et al.* 2016; Dalla Costa *et al.* 2020). The EEG represents the functional electrical activity of the brain and is considered a more accurate and reliable indicator of brain function/dysfunction than behavioural or brainstem reflexes. Generally, a normal EEG in a conscious mammal will include low-amplitude, high-frequency alpha (8 to 12 Hz) and beta (12–30 Hz) waveforms but after brain trauma-induced unconscious, the waveforms shift to predominately high-amplitude, low-frequency delta waves (0.5 to 4 Hz). Brain death occurs when the EEG flatlines or becomes ‘isoelectric’ (Grandin 2020). There is a ‘transition zone’ between the state of being fully conscious (alive) and brain death (dead) that is difficult to determine. It occurs somewhere between observed behavioural unresponsiveness and isoelectric EEG and happens at some point during the loss of high-frequency waveforms (alpha and beta) and the shift to delta and theta waveforms (Grandin 2020). The EEG is a representation of the activity of the brain that provides a more direct indication of the disruption of brain function and in some situations is considered a more reliable indicator (than behaviour) of when undoubted unconsciousness is present (Gibson *et al.* 2019). Death in animals shot with a captive-bolt gun can be caused by a lack of oxygen to the brain (associated with cardiac and/or respiratory arrest or exsanguination when used as a secondary slaughter or euthanasia method) or by brain damage caused by the stun (especially in smaller animals) (Terlouw 2020).

The objective of this current project was to assess the efficacy of captive-bolt devices for the euthanasia of pouch young during the commercial harvesting of kangaroos. We initially reviewed existing data on the performance characteristics (bolt velocity, kinetic energy, penetration depth etc) for a range of potentially suitable captive-bolt devices. When data were not already available for some devices, we conducted relevant testing of mechanical performance in the laboratory. We collated this information and then determined the suitability for field use of a small selection of suitable penetrating and non-penetrating captive-bolt devices. Four different models of captive-bolt device were subsequently tested in field experiments on live animals. Stunning and death was assessed using behavioural and electroencephalographic (EEG) indices, while damage to the skull and brain was also assessed by examining gross pathology, post mortem.

## Materials and methods

### Mechanical performance of captive-bolt devices

The performance characteristics (bolt velocity, kinetic energy, penetration depth) of a range of potentially suitable captive-bolt devices were reviewed. Where data had not yet been published, the devices were tested in the laboratory. Bolt velocity was measured with a custom-built velocity meter (Solutions for Research Ltd, Silsoe, Beds, UK) using methodology described in Gibson *et al.* (2015b) and previously reported in Sharp *et al.* (2015). The meter measured velocity of the bolt as it transects a series of seven infrared light-emitting diodes (LEDs). The LEDs are positioned 4-mm apart and the time taken to transect consecutive LEDs was used to calculate the bolt velocity.

The CBDs were fired either 15 or 40 times for velocity assessment using the meter. Peak velocity was taken as the highest mean velocity recorded. Peak velocity of the bolt was recorded and then used to calculate the kinetic energy of the bolt (*Kinetic energy = [0.5 × m] × v^2^*; where *m* = mass of the bolt [kg] and *v* = peak velocity [m s^–1^]).

Bolt weights were provided by the manufacturer or measured on a precision balance. Penetration depth or maximum bolt travel distance was provided by the manufacturer (TED, CASH® SAT, Zephyr EXL, Blitz Schlag, Kleiner Blitz and Blitz Kerner) or measured by firing of the captive-bolt guns into ballistics gelatine moulds (CASH® Special, Blitz Kerner) or modelling clay (Blitz Schlag). The ballistics gelatine was prepared according to Fackler and Malinowski (1988).

### Field testing of captive-bolt devices

The project was conducted in accordance with the Australian Code for the Care and Use of Animals for Scientific Purposes, 8^th^ edition (NHMRC 2013) with approval from the NSW Department of Primary Industry’s Animal Ethics Committee (Animal Research Authority number OAEC-0549) and the Royal Veterinary College Clinical Research Ethical Review Board (URN 2023 2194-3). A scientific licence (licence number SL102731), issued by the NSW Department of Planning and Environment under the Biodiversity Conservation Act 2016, was also granted for the project.

Field studies to test the CBDs on live kangaroo pouch young were conducted at the University of New South Wales, Fowlers Gap Research Station, located north of Broken Hill in Western NSW. Female red kangaroos, with large visible pouch young and no observable young at foot, were killed at night with an accurately placed head shot performed by an individual professional kangaroo shooter as per the Code. Immediately after the females were confirmed dead, the partially furred to fully furred young were taken from the pouch and placed in a cotton bag/pillowcase (which acted as an artificial pouch). The bag was suspended from a timber dowel mounted horizontally inside a plastic crate. The bottom of the crate was lined with a thick piece of foam to minimise vibration and noise, and a lid containing airholes was placed on top. Each individual joey was held within their own bag during transportation to a central CBD testing field site, which was no more than 10 km (mean [± SD] distance: 3.46 [± 2.18] km; range: 0.12–9.49 km) from where they were caught. The joeys were removed from the box, but kept within the bag/pillowcase, one at a time for testing with the CBD or concussive blow. The mean (± SD) time from capture to processing was 51 (± 27.5) min. Morphometric measurements (pes length, tail length, head length, leg length and bodyweight) were taken after the young had been euthanased and death confirmed. Pouch young age was determined based on previous studies that examined the relationship between known age and tail length for red kangaroos (Sharman *et al.* 1964).

Based on a combination of performance characteristics (e.g. kinetic energy, bolt penetration depth; Gibson *et al.* 2015b); practicality and ease of use in a field setting (e.g. compressed air is not feasible); suitability for use on small animals such as pouch young; availability; and cost, four types of CBDs were chosen to be tested. These devices were:

- *CASH® SAT (Small Animal Tool)* (Accles & Shelvoke, Sutton Coldfield, UK) (non-penetrating) (with brown activators) – powered by .22 blank 1.0 gr cartridge, suitable for small animals and birds;
- *Blitz Schlag* (turbocut Jopp GmbH, Bad Neustadt an der Saale, Germany) (with green activators) (non-penetrating) – powered by 9 mm/.38 calibre blank centre-fire cartridge, suitable for small animals and birds;
- *Blitz Kerner* (turbocut Jopp GmbH, Bad Neustadt an der Saale, Germany) (with green activators) (penetrating) – powered by 9 mm/.38 calibre blank centre-fire cartridge, suitable for a range of species including larger livestock; and
- *TED (Turkey Euthanasia Device)* (Bock Industries, Phillipsburg, PA, USA) (non-penetrating) – powered by propane fuel canister, suitable for poultry, rabbits, piglets (under 10 kg) and goat kids (under 4 kg).

To determine if cartridge power loads varied, the cartridges used for the CASH® SAT, Blitz Kerner, and Blitz Schlag were weighed on a precision balance prior to and after the shooting.

All four CBDs were initially tested on a small number of live kangaroo young (n = 5 per device) (Stage 1) and were then ranked from one to four based on the characteristics of efficacy, ease of use, operator safety, durability, and ease of maintenance. The three highest-ranked, captive-bolt devices were chosen for further live animal testing (Stage 2).

### Application of euthanasia methods

#### Captive-bolt devices

Red kangaroo pouch young (joeys) (n = 49), mean (± SD) weight: 2.56 (± 1.10) kg; range: 0.64–5.36 kg, and mean age of 212 (± 20.9) days; range: 156–248 days, were placed on a pillow while still been held in the artificial pouch; the head was exposed allowing placement of a blindfold over the eyes. Pre-treatment EEG signals were recorded with the head physically restrained by an operator to minimise movement artefact. After pre-treatment EEG recording, joeys were shot with captive bolt on the top (or crown) of the head at the midline aiming slightly back towards the base of the jaw (Sharp *et al.* 2015). One individual operator, a member of the research team, performed all the shooting with the CBDs.

#### Manual blunt force trauma to the head

Red kangaroo pouch young (joeys) (n = 6), mean weight: 3.28 (± 1.46) kg; range: 1.16–5.40 kg, and mean age of 219 (± 21.7) days; range: 177–234 days, were killed by manual blunt force trauma to the head (also referred to as manually applied concussive blow) by a single experienced kangaroo shooter holding the animal by its rear legs and striking the top of the head against the flat metal tray of the back of a utility vehicle set up for harvesting. This method (and slight variations) is the current main euthanasia method used by commercial kangaroo shooters for partially to fully furred pouch young (Sharp 2015). Pre-treatment EEG signals were recorded with the head physically restrained by an operator to minimise movement artefact. After pre-treatment EEG recording, the electrodes were removed, and the blunt force trauma was applied. The operator, holding both rear legs in his hands and supporting the joey’s body against his shoulder, moved into position behind the tray of the vehicle. The joey was swung downwards with a continuous single strong and fluid motion with the head impacting against the tray of the vehicle at the end of the swing arc. The intended impact site was the top of the cranium between the ears. After the concussive blow to the head, the joey was immediately transferred to a table behind the operator for post-treatment behavioural assessment of brain function (within 3 s), placement of EEG electrodes and recording of EEG. The mean interval from impact to the beginning of EEG recording and assessment was 24.3 (± 3.7) s.

### Behavioural assessment of brain function

The behaviour of each joey was observed based on an ethogram (Table 1) devised to focus on measures described by Fletcher *et al.* (2025), Gibson *et al.* (2015a) and Terlouw *et al.* (2016) that are indicative of unconsciousness and death in live animals subjected to shooting with a CBD and manual blunt force trauma. The presence or absence of rhythmic breathing, palpebral and corneal reflex, spontaneous eye blinking, jaw tension, gasping and convulsions were evaluated immediately after application of the euthanasia method by two members of the research team, with a third member recoding the observations manually. The measures were then continuously monitored in the same order for all animals across all treatments for a minimum period of 5 min, with any significant changes noted and actions taken to euthanase when necessary. If joeys showed any sign of consciousness or risk of returning to consciousness after stunning, they were euthanased using an intravenous, intraperitoneal or intracardiac injection of pentobarbitone (Lethabarb ® Virbac, Australia) at a dose rate of 150 mg kg^–1^. The stunning method was considered effective when rhythmic breathing ceased, there were no brainstem-related reflexes and the EEG indicated that brain activity had become isoelectric, consistent with death. As stated above, even when unconsciousness and/or death was established, monitoring continued for a minimum of 5 min for all joeys. Additional information on blood extravasation (bleeding from the mouth, ears, and nostrils) was also recorded.

**Table 1.** Ethogram with indicators of consciousness and unconsciousness (based on Fletcher *et al.* 2025, Gibson *et al.* 2015a; Terlouw *et al.* 2016)

Behaviour	Description and interpretation
Rhythmic (normal) breathing	Regular rhythmic movement of the thoracic/diaphragm area where the ribs move in an out at least twice Indicates medullary function and potential for oxygenated blood to reach the brain which could lead to resumption of consciousness
Palpebral reflex	Involuntary blink reflex when the medial canthus and/or eyelash is stimulated Presence indicates brainstem function, which could support consciousness
Corneal reflex	Involuntary blink reflex when corneal is stimulated Presence indicates brainstem function, which could support consciousness
Spontaneous eye blinking	Opens/closes eyelid without stimulated Indicator of brainstem function, presence could indicate brainstem function and/or coordinated blinking
Jaw tension	Resistance to manual manipulation of the jaw Can indicate coordinated conscious muscle tone
Gasping	Intermittent, spasmodic, forceful intake of breath with the mouth open, that is not rhythmic Can indicate cerebral ischaemia and/or hypoxia
Convulsions	Tonic convulsions: stiffening/rigidity of muscles Clonic convulsions: uncontrolled involuntary kicking movement Clonic convulsions are hypothesised to occur due to the loss of descending inhibitory control from the cerebral cortex, allowing brainstem and spinal motor circuit activity to be unregulated.

### EEG recording and assessment

EEG was recorded using three 27-gauge stainless steel subdermal electrodes (Neuroline Subdermal, Ambu Inc, Glen Burnie, MD, USA), placed as a three-electrode montage in the scalp skin with the active (non-inverting) left of midline in-line with the back of the eyes; reference (inverting), over the left caudal aspect of the frontal bone (top of head) in-line with the front of the ears; and ground electrode caudal to the poll. EEG signals were amplified and filtered with an analogue filter (Dual Bio Amp, ADInstruments Ltd, Sydney, Australia) with low- and high-pass filters of 300 and 0.1 Hz, respectively. The signals were digitalised (1 kHz) with a 4/35 PowerLab (ADInstruments Ltd, Sydney, Australia) digital-to-analogue converter and recorded on an Apple MacBook for off-line analysis. Mean interelectrode impedance was 1.37 (± 0.32) kΩ and ranged between 0.95 and 3.00 kΩ (MkIII Checktrode, UFI, Morro Bay, CA, USA). Each animal acted as their own control with comparisons made between pre- and post-treatment EEG waveforms.

EEG segments contaminated by over- and under-scaling (signal drift), large single spikes, electromyography (muscle activity) or movement artefact were manually rejected from analysis using LabChart 8.1.24 (ADInstruments Ltd). All waveforms were digitally filtered with a passband of 1 to 30 Hz, and traces were inspected visually and compared with baseline using the classification systems developed by Gibson *et al.* (2009b). They were classified into one of five categories previously published in the literature (Gibson *et al.* 2019): (1) movement artifact; (2) normal-like active EEG; (3) transitional EEG; (4) high-amplitude low-frequency (HALF) EEG; and (5) isoelectric EEG. Briefly, normal-like EEG represents an activity that is similar in amplitude and frequency to the baseline period. Transitional EEG was classified as suppressed activity of having either an amplitude of less than half of that of the pre-treatment EEG and/or depressed high-frequency activity. HALF EEG was a waveform of high amplitude and low frequency. Isoelectric EEG was classified as a trace with an amplitude of < 1/8 (12.25%) of that of normal pre-stunning EEG with little or no low-frequency components.

### Pathology assessment

After confirmation of death, and collection of morphometric data, the heads were removed and stored upright in individually sealed clear plastic bags for freezing. Heads were frozen at –4°C for a minimum of 48 h prior to assessment of gross pathology. The frozen skulls were sawn with a reciprocating saw down the medial/sagittal plane of the head with the brain *in situ.* When the shot/impact site could be identified in frozen heads, they were sawn through the injury site, while the remaining heads were sawn on midline. Each sawn section was carefully polished with a warm, damp towel to remove potential saw artefacts that may have arisen from sawing. Twenty-four hours later the heads were defrosted, and brains were extracted. The skull and brains were examined both *in situ* (frozen and defrosted) and after extraction (defrosted), for shot/impact position, bolt penetration angle and trajectory, skull and skin thickness, and fractures (including number of radiating and depressed fractures). Shot impact site was measured as variations rostrally/caudally and left/right from the reference point, which was the crossover of lines projected from the middle of the eye to the middle of the base of the opposite ear.

The brains were examined for gross damage to specific neurological structures, haemorrhage, and displacement of tissues. Data from the left and right hemispheres were pooled to aid analysis. Severity of tissue damage to specific brain regions (thalamus/hypothalamus, midbrain, pons, medulla, cerebellum and frontal, parietal, temporal and occipital lobes) was subjectively assessed as none (0%), mild (1–20%), moderate (21–49%) and severe (≥ 50%) (Gibson *et al.* 2012, 2015b,c). Haemorrhage over the entire brain surface was subjectively assessed using the same severity scale as used for brain regions.

### Statistical analysis

We used logistic regression to determine if captive-bolt type (penetrating vs non-penetrating) was related to the likelihood of failure to produce enduring unconsciousness. We used success/failure of enduring unconsciousness as a binary response variable and captive-bolt type as the predictor variable. The predictor variable captive-bolt type (penetrating = 0 or non-penetrating = 1) was a factor, and the response variable was a binary outcome describing the need to euthanase an animal after the captive-bolt shot (defined as a failure) (euthanased = 1, not euthanased = 0). In this analysis, the ideal situation is that the young do not require euthanasia after shooting with the captive-bolt gun. Positive values for the predictor variable indicate that more pouch young require euthanasia after shooting with the non-penetrating captive bolt treatment, while negative values indicate the opposite. The analysis was performed using the statistical programming language R (R Core Team 2024), using the probabilistic programming language Stan (Stan Development Team 2024a) via the R package rstan (Stan Development Team 2024b). We used weakly informative priors, which took default values: normal (0, 2.5) for the intercept and normal (0, 5) for the coefficient of captive-bolt type.

The rate of success of effective stunning for each captive-bolt device and 95% credible intervals was calculated using Stan and R. The analysis used the number of trials and successes (effective stuns not requiring euthanasia), to estimate the binomial probability of an effective stun for each bolt type. We used published studies of the effectiveness of CBDs (Sutherland *et al.* 2016; Grist *et al.* 2018a,b,c; Hampton 2019) to estimate an informative prior for theta (the success rate), which was beta (98, 2).

## Results

### Mechanical performance of captive-bolt devices

The performance characteristics of all potentially suitable CBDs, including the four devices chosen for field testing with this study, are presented in Table S1 (Supplementary material). The mean peak velocity (47.4 [± 0.7] m s^–1^) and mean kinetic energy (234.8 [± 7.2] J) were highest for the Blitz Kerner device and of the four tested devices, the CASH® SAT had the lowest mean peak velocity (29.3 [± 1.0] m s^–1^) while the TED delivered the lowest mean kinetic energy (28.4 [± 0.4] J).

### Field testing of captive-bolt devices

During Stage 1 of testing, we observed variation in the efficacy and useability for the four different CBD models. All animals shot with the Blitz Kerner (n = 5) and the TED (Turkey Euthanasia Device) (n = 5) devices lost consciousness and stopped breathing without recovery. One of the five animals shot with the Blitz Schlag returned to rhythmic breathing and needed to be euthanased with sodium pentobarbitone. Two of the five animals shot with the CASH® Small Animal Tool (SAT) also returned to rhythmic breathing and needed to be euthanased with sodium pentobarbitone. One of these also had normal-like active EEG.

There was only a low level of variability in the power loads of the cartridges used in the CASH® SAT, Blitz Kerner and Blitz Schlag devices prior to and after shooting (see Table S2; Supplementary material), indicating that cartridge propellant fill was consistent and not a contributing factor to the failed stuns.

With regard to useability, the Blitz Kerner, Blitz Schlag and TED CBDs were found to be ergonomic and easy to use, however the CASH® SAT felt awkward to hold with both a single and two-handed grip and the trigger button was difficult to press (although hand size may have been a contributing factor). The TED required the least maintenance, and given its construction and mode of use, it was likely to be the safest, however we deemed it to be the least durable of the four CBDs tested. Both the Blitz Kerner and Blitz Schlag required thorough cleaning at the end of each session. This is recommended by the manufacturer to remove powder residue and prevent corrosion regardless of whether they are fired only once or multiple times.

Table 2 presents an overview of the important performance characteristics, prices, and the results of our ranking of the four CBDs tested in Stage 1 based on a range of criteria including efficacy and useability. In summary, the Blitz Kerner and the TED had no failed stuns during Stage 1, while the Blitz Kerner and the Blitz Schlag were the easiest to use. The CASH® SAT is a solid, durable device however, since it failed to cause a complete stun in two out of five animals and was not ergonomic to use, it was not tested any further. The other three devices were tested on live animals in Stage 2, and the results are presented in the following section. Note that the animals tested in Stage 1 (n = 5 for each device) are included in the total number of animals tested.

**Table 2.** Ranking of four captive-bolt devices after initial testing at Stage 1. The devices were ranked from 1 (best) to (4) worst based on: effectiveness at stunning red kangaroo joeys (*Osphranter rufus*) (efficacy); ease of handling and application (useability); robustness for field use (durability); and extent of upkeep required (ease of maintenance). Each device was tested on five live red kangaroo joeys (n = 20)

Captive bolt model	Mean (± SD) peak velocity (m s^–1^)	Mean (± SD) kinetic energy (J)	Stage 1 test efficacy	Stage 1 test useability	Operator safety	Durability	Ease of maintenance	Price (AUD)
Blitz Kerner(penetrating)	47.4 (± 0.7)	234.8 (± 7.2)	1	1	4	2	3	$450($80 for 50 activators)
Blitz Schlag (non-penetrating)	32.6 (± 2.9)	131.8 (± 1.0)	2	1	2	2	3	$486($80 for 50 activators)
CASH SAT (non-penetrating)	29.3 (± 1.0)	77.1 (± 5.0)	3	3	3	1	2	$2,428(with 1,000 cartridges)
TED (non-penetrating)	30.4 (± 0.2)	28.4 (± 0.4)	1	2	1	3	1	$2,750

### Behavioural assessment of brain function

All animals shot with the penetrating CBD (Blitz Kerner; n = 20) did not show any behavioural indicators of consciousness after shooting. Rhythmic breathing, palpebral and corneal reflexes, jaw tension and spontaneous eye blinking were all absent and death was confirmed during the five minutes of observation.

Seven of the 29 animals shot with non-penetrating CBDs (Blitz Schlag; n = 2; CASH® SAT n = 2; and TED n = 3) displayed either indicators of consciousness or a risk of returning to consciousness after shooting and were euthanased using an injection of barbiturate. One joey (ID001, BS1), shot with the Blitz Schlag, had normal rhythmic breathing immediately after stunning, but palpebral and corneal reflexes, jaw and neck tension and spontaneous eye blinking were all absent. For the other six animals, rhythmic breathing, palpebral and corneal reflexes, jaw tension and spontaneous eye blinking were all absent immediately after stunning however they returned to rhythmic breathing in the five-minute observation period.

All other animals shot with the three non-penetrating devices did not show any behavioural indicators of consciousness after shooting.

Animals killed with manual blunt force trauma to the head, (n = 6) did not show any behavioural indicators of consciousness after the concussive trauma. Rhythmic breathing, palpebral and corneal reflexes, jaw tension and spontaneous eye blinking were all absent and death was confirmed during the five minutes of observation. Five of the six animals presented bleeding from the nose, three bleeding from the ear(s) and two had prolapsed eyeballs. Five also exhibited tonic/clonic convulsions immediately after stunning. With one animal (Joey 056 BF5), the site of impact was to the nose/muzzle rather than the head. The kangaroo shooter recognised immediately that this was a missed hit and, without hesitation, applied a second blow which struck the head in the correct position. For the other five animals a single blow was applied, and the site of impact was the head.

### EEG recording

The subjective analysis is the visual characterisation of waveform morphology. It provides duration, and by proxy, time to onset of different EEG states. The analyses were limited to a 5 min (300 s) period as beyond this point all recordings were isoelectric. Note that for the seven animals that were euthanased with a lethal injection, where possible we have added a marker (asterisk) for time of delivery of pentobarbitone in the figures, however we did not record the time of injection for most animals. Based on movement artefact on the EEG traces, the lethal injection occurred after five minutes when the EEG was already isoelectric for the remaining animals.

Seventeen (85%), one (20%) and two (17%) of the joeys shot with the Blitz Kerner, CASH® SAT and TED CBD’s, respectively, had periods of ‘no data’ recorded immediately after the shot, which was due to physical displacement of EEG electrodes by the action of captive-bolt shooting. When electrodes were observed as being displaced, they were immediately replaced. There were no periods of ‘no data’ recordings with the Blitz Schlag. For the Blitz Kerner, CASH® SAT and TED, the mean duration of no recorded data was 18.9 (± 16.1); (range 5–67), 8 (n = 1) and 5.5 (± 6.4); (range 1–10) s, respectively.

**Figure 1.**
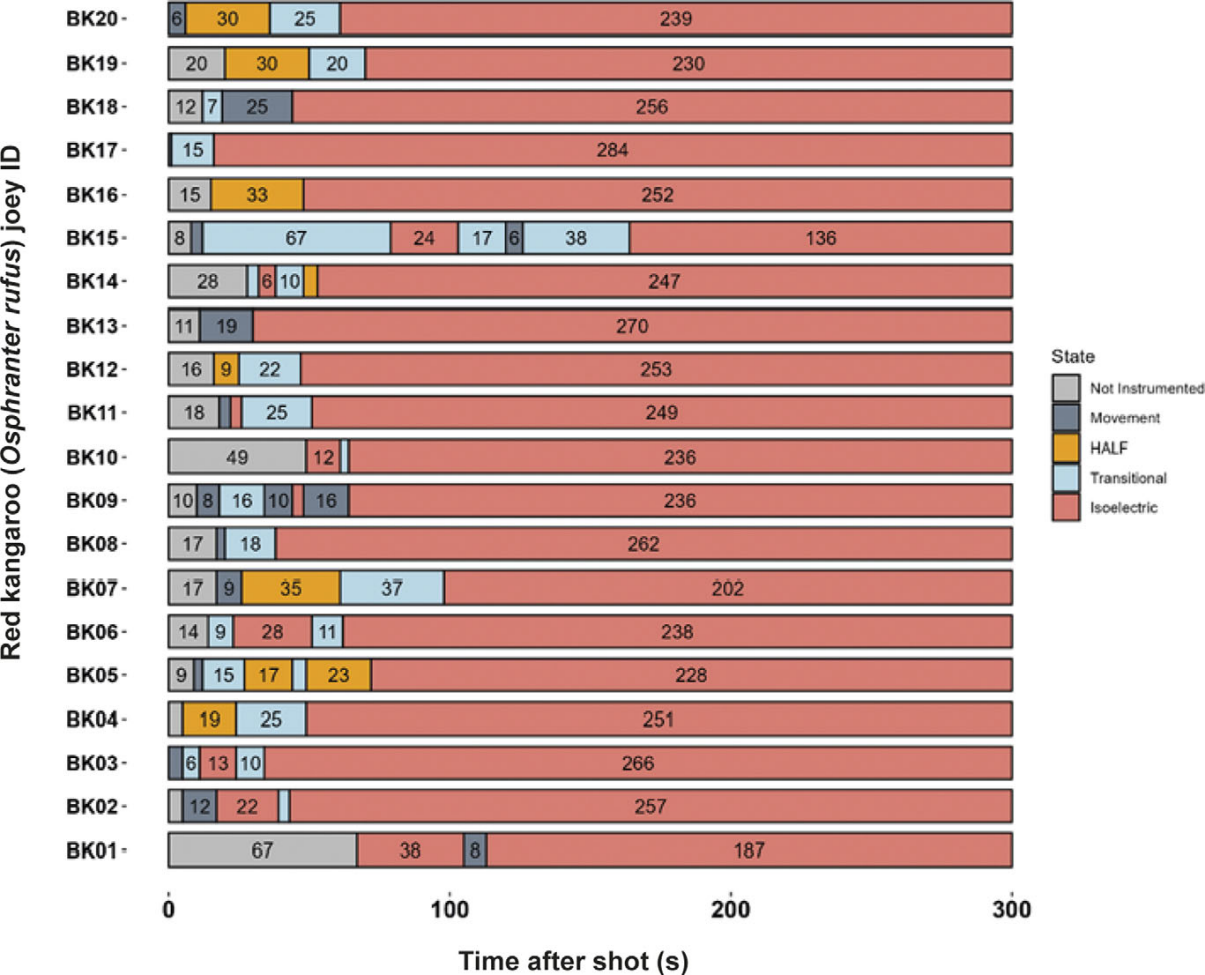
Characteristics of electroencephalogram (EEG) waveforms in individual red kangaroo (*Osphranter rufus*) joeys (n = 20) shot (time-point 0) with the Blitz Kerner (BK). Light grey bars represent periods when no data were collected due to joeys not being instrumented; dark grey movement artefact; blue transitional EEG; pink isoelectric EEG; and orange high amplitude low frequency (HALF). Numbers within bars represent duration of the EEG states (in s).

**Figure 2.**
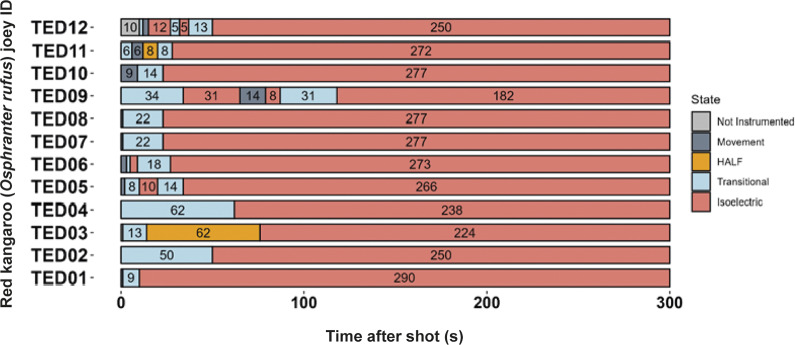
Characteristics of electroencephalogram (EEG) waveforms in individual red kangaroo (*Osphranter rufus*) joeys (n = 12) shot (time-point 0) with the Turkey Euthanasia Device (TED). Light grey bars represent periods when no data were collected due to joeys not being instrumented; dark grey movement artefact; blue transitional EEG; pink isoelectric EEG; and orange high amplitude low frequency (HALF). Numbers within bars represent duration of the EEG states (in s).

**Figure 3.**
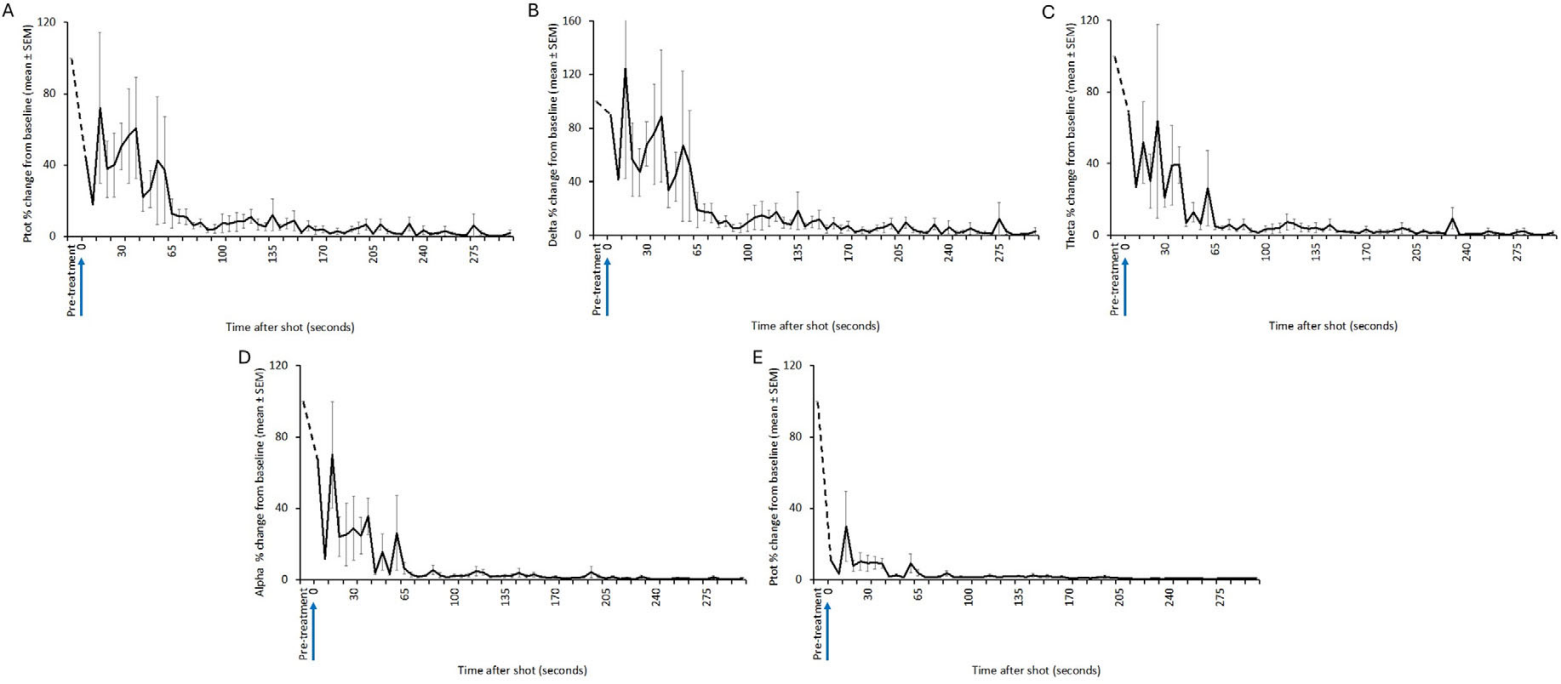
Mean (± SEM) total power (Ptot) (A), delta (B), theta (C), alpha (D) and beta (E) frequency bands of the electroencephalogram (EEG) of red kangaroo (*Osphranter rufus*) joeys (n = 20) before and after shooting with the Blitz Kerner (shot at 0 s). Note periods artefact (movement and/or displacement of electrodes) are indicated by dashed lines.

**Figure 4.**
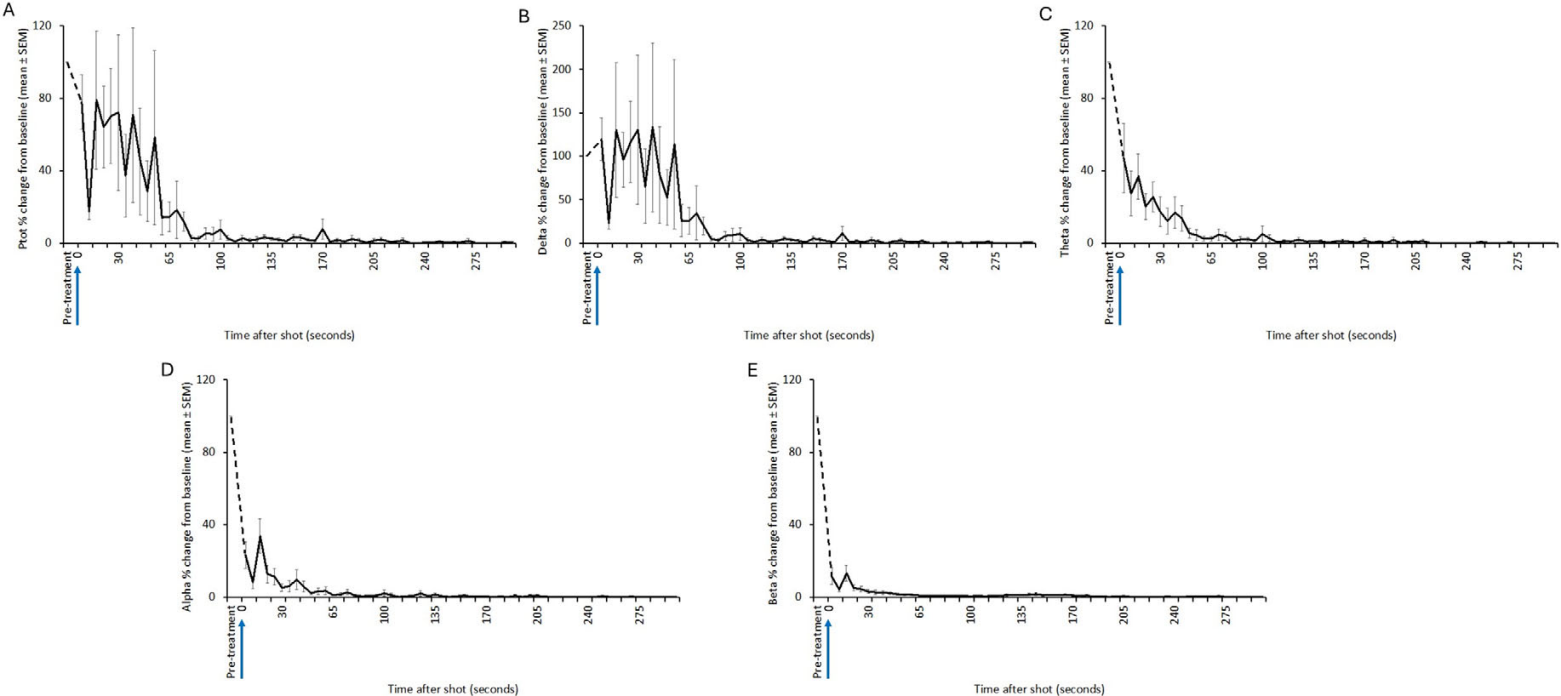
Mean (± SEM) total power (Ptot) (A), delta (B), theta (C), alpha (D) and beta (E) frequency bands of the electroencephalogram (EEG) of red kangaroo (*Osphranter rufus*) joeys (n = 12) before and after shooting with the TED (shot at 0 s). Note periods artefact (movement and/or displacement of electrodes) are indicated by dashed lines.

All animals shot with the Blitz Kerner (n = 20) and TED (n = 12) had patterns of EEG activity that were inconsistent with consciousness (Figures 1 and 2, respectively). Generally, this was characterised by periods of transitional and HALF EEG, before changing to an isoelectric waveform. This pattern was also seen in the spectral analysis with a decrease in power following the shot (ptot), particularly in the alpha and beta frequency bands (Figures 3 and 4). In some joeys there was increased power in the delta and theta bands, which was also seen as increased HALF activity in the subjective analysis. These periods of activity in the Blitz Kerner and TED shot joeys was associated with a pronounced decrease in alpha and beta power.

**Figure 5.**
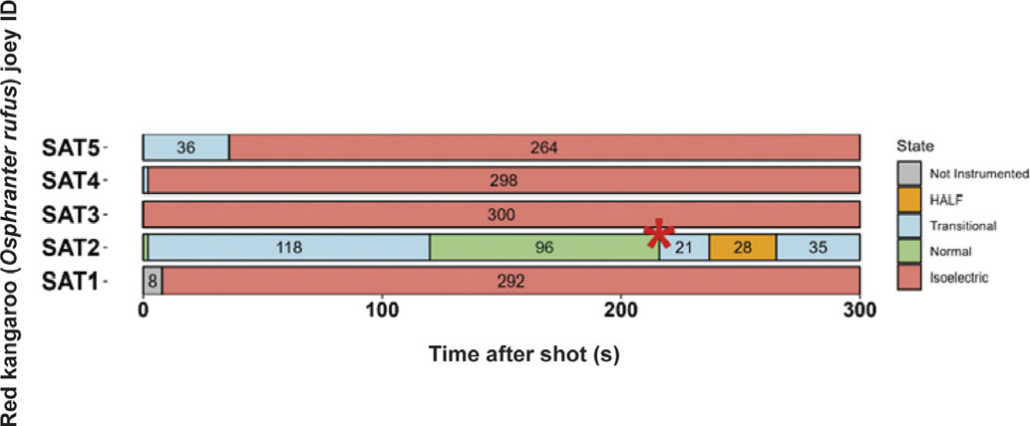
Characteristics of electroencephalogram (EEG) waveforms in individual red kangaroo (*Osphranter rufus*) joeys (n = 5) shot (time-point 0) with the CASH Small Animal Tool (SAT). Light grey bars represent periods when no data were collected due to joeys not being instrumented; blue transitional EEG; pink isoelectric EEG; orange high amplitude low frequency (HALF); and green bars representing normal-like active EEG (non-complete concussion). The red asterisk denotes the application of pentobarbitone sodium to SAT2, EEG became isoelectric at 216 s. Numbers within bars represent duration of the EEG states (in s).

**Figure 6.**
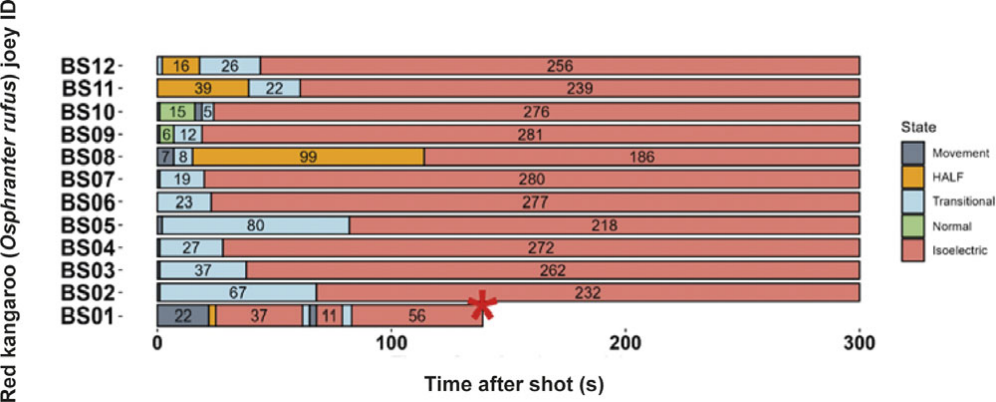
Characteristics of electroencephalogram (EEG) waveforms in individual red kangaroo (*Osphranter rufus*) joeys (n = 12) shot (time-point 0) with the Blitz Schlag (BS). Light grey bars represent periods when no data were collected due to joeys not being instrumented; dark grey movement artefact; blue transitional EEG; pink isoelectric EEG; orange high amplitude low frequency (HALF); and green bars representing normal-like active EEG (non-complete concussion). Red asterisk denotes the application of pentobarbitone sodium to BS1, EEG recording was stopped at this point. Numbers within bars represent duration of the EEG states (in s).

**Figure 7.**
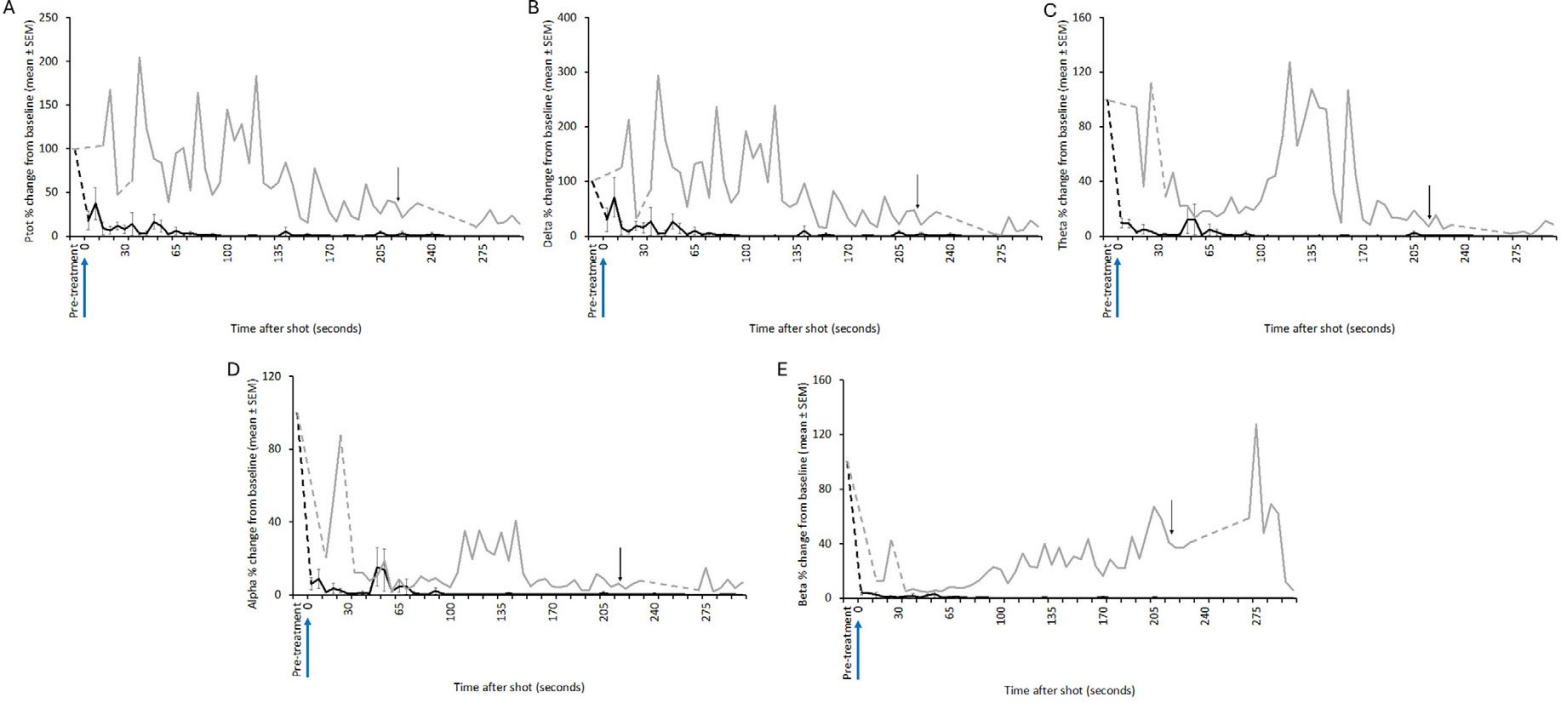
Mean (± SEM) total power (Ptot) (A), delta (B), theta (C), alpha (D) and beta (E) frequency bands of the electroencephalogram (EEG) of red kangaroo (*Osphranter rufus*) joeys (n = 5) before and after shooting with the CASH Small Animal Tool (shot at 0 s). Joey SAT2 is indicated by the grey line in figures A, B, C, D and E, the black arrow is the point of administration of pentobarbitone sodium for this joey. Note periods artefact (movement and/or displacement of electrodes) are indicated by dashed lines.

**Figure 8.**
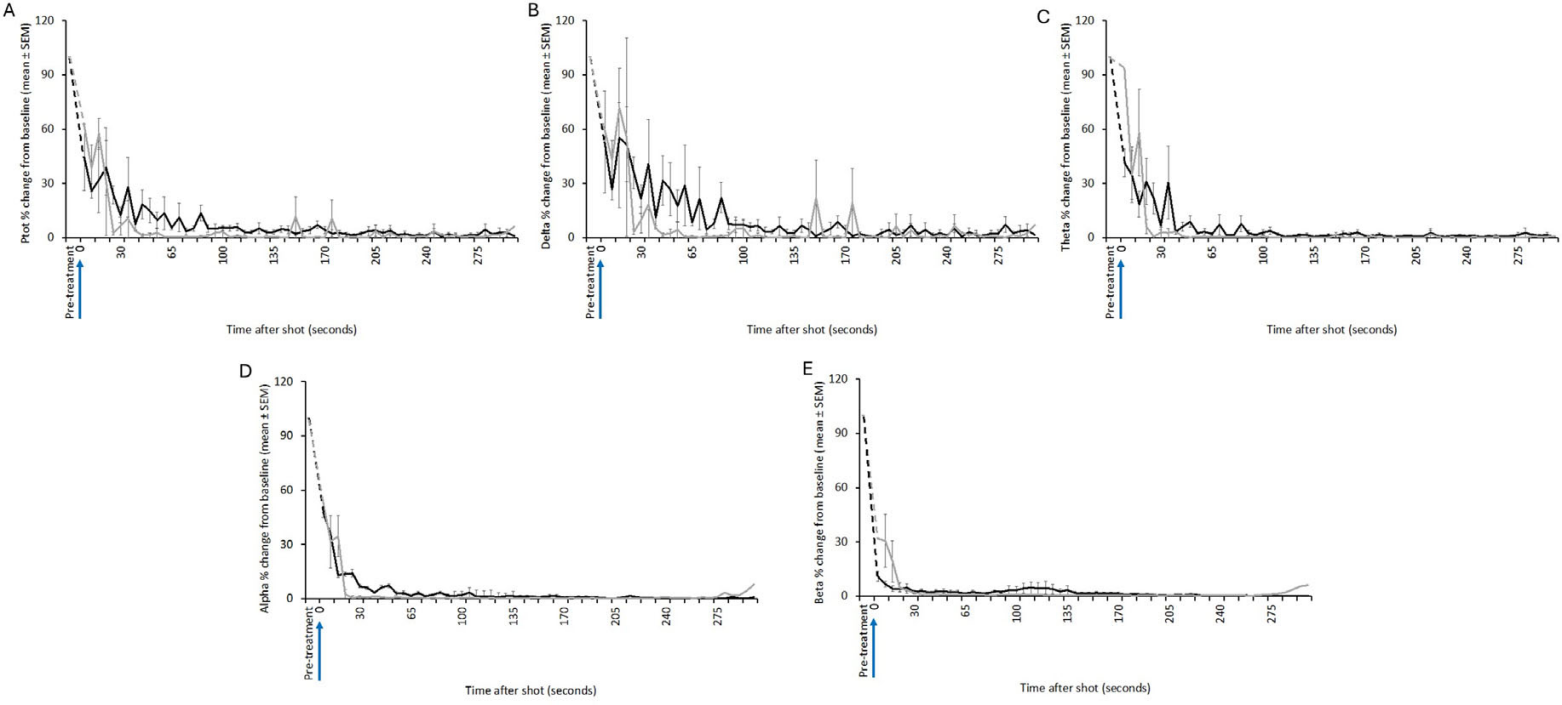
Mean (± SEM) total power (Ptot) (A), delta (B), theta (C), alpha (D) and beta (E) frequency bands of the electroencephalogram (EEG) of red kangaroo (*Osphranter rufus*) joeys (n = 12) before and after shooting with the Blitz Schlag (shot at 0 s). The mean (± SEM) of joeys BS9 and BS10 is indicated by the grey line in figures A, B, C, D and E. Note periods artefact (movement and/or displacement of electrodes) are indicated by dashed lines.

Three joeys, one shot with the CASH® SAT (Figure 5) and two shot with the Blitz Schlag (Figure 6), had periods of normal-like active EEG after the captive bolt. Joey SAT2 had biphasic active normal-like EEG activity initially after the shot and following a 118-s period of transitional activity. The second period of active normal-like EEG lasted 96 s before changing to periods and transitional and HALF. The periods of active normal-like EEG were associated with increased power in all frequency bands, particularly beta (Figures 7 and 8). Meanwhile the two joeys, BS9 and BS10 (Figure 8) had initial periods active normal-like EEG shot (6 and 15 s, respectively), which corresponded with periods of increased power in delta, theta and beta frequency bands, before changing to transitional and then isoelectric waveforms.

**Figure 9.**
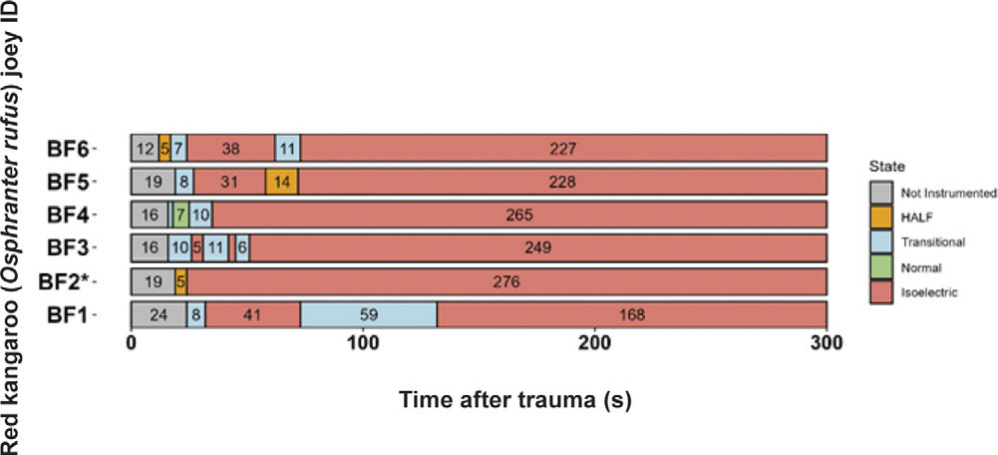
Characteristics of electroencephalogram (EEG) waveforms in individual red kangaroo (*Osphranter rufus*) joeys euthanased by blunt force trauma (time-point 0). Light grey bars represent periods when no data were collected due to joeys not being instrumented; blue transitional EEG; pink isoelectric EEG; orange high amplitude low frequency (HALF); and green bars representing normal-like active EEG (non-complete concussion). *BF2 was hit twice, 0 denotes the point of the first hit. Numbers within bars represent duration of the EEG states (in s).

**Figure 10.**
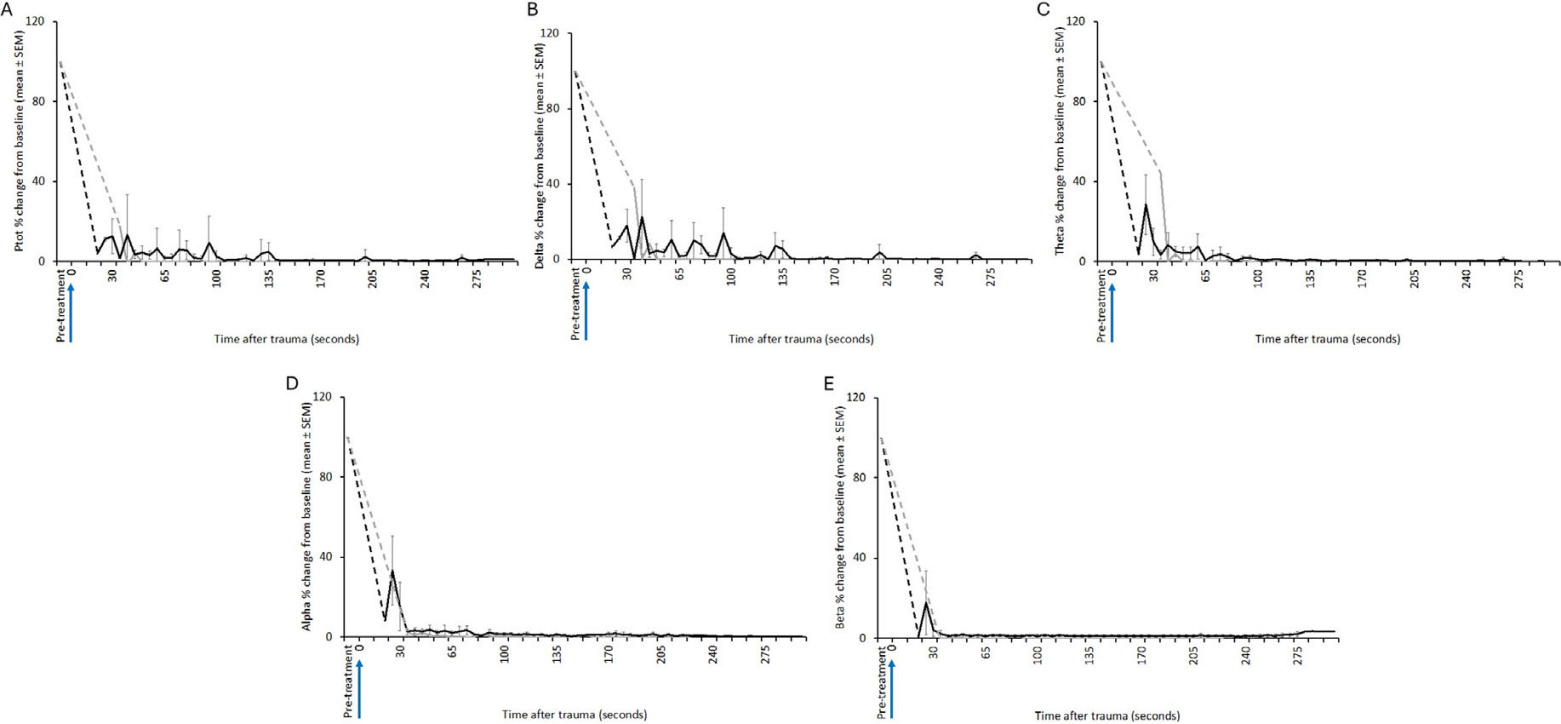
Mean (± SEM) total power (Ptot) (A), delta (B), theta (C), alpha (D) and beta (E) frequency bands of the electroencephalogram (EEG) of red kangaroo (*Osphranter rufus*) joeys (n = 6) before and after manual blunt force trauma (black line excluding BF4) (blunt force trauma at 0 s). The grey line is joey BF4. Note the period when no data were collected due to joeys not being instrumented and/or due to movement artefact is indicated by a dashed line.

All animals killed by manual blunt force trauma had periods of no recorded data, as it was impractical to have the joeys instrumented with EEG electrodes during the treatment. Immediately after contact with the flat metal tray of the vehicle, joeys were brought to the recording area, instrumented and EEG recordings commenced. The mean duration of this period was 17.7 (± 4.0); (range 12–24) s (Figure 9). Joey BF5 received two hits after being struck inaccurately (on the muzzle) with the initial hit. For this animal, time-point zero represents the first hit. One joey (BF4) had a 7-s period of active normal-like EEG activity after the hit before changing to transitional and then isoelectric (Figure 9). The period of active normal-like EEG in BF4, was characterised by a lower reduction in EEG power in the delta, theta and beta bands compared to the other joeys (Figure 10). Most animals had periods of transition and/or HALF EEG activity before becoming isoelectric.

### Shot position of the captive-bolt devices

**Figure 11.**
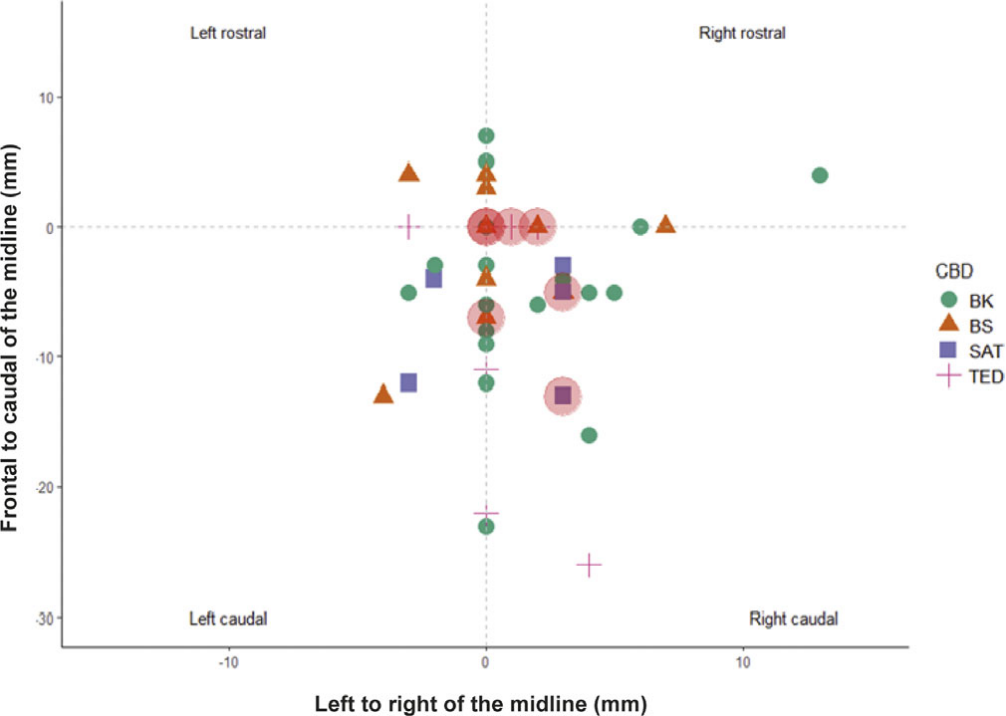
Bolt impact site for the four captive-bolt devices (CBD) (BK = Blitz Kerner, BS = Blitz Schlag, SAT = CASH Small Animal Tool, TED = Turkey Euthanasia Device) relative to the reference point on the head of red kangaroo (*Osphranter rufus*) joeys (n = 49). The reference point was the location at which imaginary (dotted) lines projected from the middle of the eye to the middle of the base of the opposite ear crossed over. The large red circles indicate the seven incomplete stuns. Note that some data-points are overlapping.

Bolt impact sites on the head varied between animals and captive-bolt types, however there were no obvious trends in entry or impact site. Shot positions ranged from 26 mm caudal to 7 mm rostral of the reference point (which was the crossover of lines projected from the middle of the eye to the middle of the base of the opposite ear) and between 4 and 13 mm left and right of midline of the skull (Figure 11).

### Pathology assessment

All animals had skull fractures from captive-bolt stunning or manual blunt force trauma. The majority had skull depression fractures with many also having linear radiating fractures from the shot depression/hole or point of impact. Animals that were killed using blunt force trauma had extensive skull fractures on both sides of the cranium with the majority extending from the region of the cerebellum to the back of the throat. They also had bone fragments in various regions of the brain. In addition, bruising to the neck was present in three of the six animals killed by blunt force trauma. For animals shot with a CBD, the CASH® SAT caused the highest mean number of skull fractures (2.61 [± 1.52] radiating fractures and 3.00 [± 1.58] depressed fractures).

For animals shot with a CBD, extrusion of brain tissue from the shot hole occurred with 63% of the shots (53% for the Blitz Kerner, 60% for the CASH® SAT, 80% for the TED and 64% for the Blitz Schlag). Mean skull thickness at the shot site was 1.94 (± 0.30) mm and mean skin thickness was 1.26 (± 0.48) mm.

All animals had some form of damage to the brain from captive-bolt stunning or manual blunt force trauma. Overall, the Blitz Kerner and CASH® SAT produced the most extensive gross damage to the thalamus, with 95 and 100% of animals, respectively, having moderate/severe damage (Table 3). The midbrain had moderate/severe damage in 90 and 100% of animals shot with the Blitz Kerner and CASH® SAT, respectively. All the animals killed using manual blunt force trauma had moderate/severe damage in the midbrain. Damage to the hindbrain was more severe in animals killed by manual blunt force trauma compared to those shot by CBDs, with severe damage caused to the pons, medulla, and cerebellum in 100% of animals. For the CBDs, the CASH® SAT caused the most amount of moderate/severe damage to the hindbrain regions (pons 60%, medulla 40% and cerebellum 100%) and the Blitz Schlag the least (pons 33%, medulla 17% and cerebellum 83%). Animals killed by manual blunt force trauma had the least amount of moderate/severe damage to the lobes of the cerebrum when compared to those shot with CBDs. Most of the damage caused by the CBDs occurred in the occipital, parietal and frontal lobes.

**Table 3.** Macroscopic gross brain damage to thalamus, brainstem, cerebellum and individual lobes of the cerebrum of red kangaroo joeys (*Osphranter rufus*) (n = 55) following mechanical stunning with Blitz Kerner penetrating captive bolt (BK) (n = 20), CASH small animal tool non-penetrating captive bolt (CASH SAT) (n = 5), TED non-penetrating captive bolt (n = 12), Blitz Schlag non-penetrating captive bolt (BS) (n = 12) and manual blunt force trauma (BFT) (n = 6). Percentage is overall proportion and number in brackets is number of animals with damage graded as none, mild, moderate and severe

Region	Severity	BK (n = 20)	CASH SAT (n = 5)	TED (n = 12)	BS (n = 12)	BFT (n = 6)
Thalamus	None	– (0)	– (0)	– (0)	8% (1)	33% (2)
	Mild	5% (1)	– (0)	17% (2)	17% (2)	33% (2)
	Moderate	5% (1)	20% (1)	33% (4)	42% (5)	17% (1)
	Severe	90% (18)	80% (4)	50% (6)	33% (4)	17% (1)
Midbrain	None	– (0)	– (0)	8% (1)	17% (2)	– (0)
	Mild	10% (2)	– (0)	25% (3)	33% (4)	– (0)
	Moderate	40% (8)	20% (1)	42% (5)	25% (3)	50% (3)
	Severe	50% (10)	80% (4)	25% (3)	25% (3)	50% (3)
Pons	None	25% (5)	20% (1)	33% (4)	33% (4)	– (0)
	Mild	20% (4)	20% (1)	42% (5)	33% (4)	– (0)
	Moderate	30% (6)	20% (1)	25% (3)	25% (3)	– (0)
	Severe	25% (5)	40% (2)	– (0)	8% (1)	100% (6)
Medulla	None	35% (7)	20% (1)	33% (4)	42% (5)	– (0)
	Mild	50% (10)	40% (2)	50% (6)	42% (5)	– (0)
	Moderate	15% (3)	– (0)	17% (2)	17% (2)	– (0)
	Severe	– (0)	40% (2)	– (0)	– (0)	100% (6)
Cerebellum	None	5% (1)	– (0)	– (0)	– (0)	– (0)
	Mild	20% (4)	– (0)	8% (1)	17% (2)	– (0)
	Moderate	25% (5)	– (0)	50% (6)	8% (1)	– (0)
	Severe	50% (10)	100% (5)	42% (5)	75% (9)	100% (6)
Frontal lobe	None	5% (1)	– (0)	– (0)	– (0)	67% (4)
	Mild	10% (2)	– (0)	8% (1)	8% (1)	– (0)
	Moderate	40% (8)	20% (1)	33% (4)	8% (1)	– (0)
	Severe	45% (9)	80% (4)	58% (7)	83% (10)	33% (2)
Parietal lobe	None	– (0)	– (0)	– (0)	– (0)	67% (4)
	Mild	5% (1)	– (0)	– (0)	– (0)	– (0)
	Moderate	10% (2)	20% (1)	17% (2)	8% (1)	– (0)
	Severe	85% (17)	80% (4)	83% (10)	92% (11)	33% (2)
Temporal lobe	None	65% (13)	40% (2)	83% (10)	50% (6)	33% (2)
	Mild	30% (6)	– (0)	8% (1)	50% (6)	33% (2)
	Moderate	5% (1)	40% (2)	8% (1)	– (0)	17% (1)
	Severe	– (0)	20% (1)	– (0)	– (0)	17% (1)
Occipital lobe	None	– (0)	– (0)	– (0)	– (0)	– (0)
	Mild	– (0)	– (0)	– (0)	– (0)	50% (3)
	Moderate	10% (2)	– (0)	8% (1)	25% (3)	17% (1)
	Severe	90% (18)	100% (5)	92% (11)	75% (9)	33% (2)

The one joey (ID001, BS1), shot with the non-penetrating Blitz Schlag, that had normal rhythmic breathing immediately after stunning, had no damage to the thalamus, midbrain, pons and medulla, mild to moderate damage in the cerebellum, frontal, temporal and occipital lobes and severe damage in the parietal lobes (see summary of macroscopic damage observed in animals with signs of incomplete concussion after shooting with a non-penetrating CBD in Table S3; Supplementary material). In addition, prior to euthanasia (with barbiturate), this particular joey had periods of transitional, movement, HALF and isoelectric activity on the EEG.

The six animals that initially stopped breathing after the shot but subsequently returned to rhythmic breathing had only mild damage to the medulla, and mild (n = 5) or moderate (n = 1) damage to the pons. Of these, only two joeys had periods of normal-like active EEG activity (SAT2 and BS10).

The four joeys that had periods of active normal-like EEG (SAT 2, BS9, BS10, and BF4) had varying degrees of damage to brainstem structures. Three that were shot with a CBD (CASH® SAT; n = 1 and Blitz Schlag; n = 2) only had mild to no damage to the pons and medulla. Meanwhile the one joey (BF4) dispatched by blunt force trauma that had active normal-like EEG, had severe damage to the midbrain, pons, and medulla but only mild damage to the thalamus.

Overall, animals that were successfully stunned using the non-penetrating CBDs had more moderate/severe damage to the thalamus (86%), pons (41%), medulla (27%) and cerebellum (96%) when compared to the seven animals that had signs of incomplete concussion after shooting with non-penetrating CBDs (thalamus 70%, pons 15%, medulla 0% and cerebellum, 70%). (see summary of macroscopic damage observed in animals successfully stunned with a non-penetrating CBD in Table S4; Supplementary material).

### Effectiveness of captive-bolt devices

The fitted Bayesian logistic regression model comparing captive-bolt gun type with the likelihood of failure showed good convergence. The 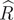 values were all equal to 1.0, and the effective sample sizes were all greater than 1,600, indicating that the chains were long enough to reach convergence. Monte Carlo standard errors were uniformly low, with the largest for both the intercept and coefficient being 0.050. Graphical evaluation of the trace plots indicated that the convergence of the intercept and coefficient had stabilised, and the chains were well mixed.

The odds ratio of the posterior mean for the captive-bolt type was 40.9, indicating the odds of causing irreversible unconsciousness after shooting with a non-penetrating captive-bolt were much lower than with a penetrating captive bolt. Simply put, the penetrating captive-bolt device has a much greater probability of rendering a pouch young irreversibly unconscious compared with a non-penetrating captive-bolt device.

The corresponding 95% credible interval for the odds ratios [2.35–6,059] indicates that there is a large degree of uncertainty in the ‘true’ value. Still, these results are consistent with the conclusion that the penetrating captive-bolt device outperformed the non-penetrating captive bolts.

In the analysis of the effective stunning rate for each captive-bolt type (Table 4), the 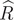 values were all equal to 1.0, and the effective sample sizes were all greater than 2,500, indicating that the chains had converged. Monte Carlo standard errors were uniformly low. Graphical evaluation of the trace plots indicated that the convergence of the intercept and coefficient had stabilised, and the chains were well mixed.

**Table 4.** Posterior means of effective stunning probability (theta) for each captive-bolt device and blunt force trauma in individual red kangaroo (*Osphranter rufus*) joeys. The table reports the standard deviation (SD) of the estimated effective stunning rate along with the lower and upper bounds of the 95% credible interval. A minimum credible threshold of 0.95 was used as the threshold for effective stunning, and only one captive-bolt device (Blitz-Kerner) exceeded this level

Captive-bolt type	Theta	SD	Lower credible interval	Upper credible interval
Blitz Kerner	0.98	0.01	0.953	0.998
Blitz Schlag	0.96	0.02	0.922	0.990
TED	0.96	0.02	0.910	0.985
CASH SAT	0.96	0.02	0.918	0.989
Blunt force trauma	0.98	0.01	0.948	0.998

Only one captive-bolt device exceeded the minimum threshold of 95% effective stun rate, and that was the Blitz Kerner. The mean effective stunning rate for the Blitz Kerner was 0.98 [0.953–0.998, 95% CI]. For all other CBDs, although the estimated mean rates exceeded 0.95, the lower credible intervals were below the 0.95 threshold, at 0.922, 0.910, and 0.918 for the Blitz Schlag, TED, and CASH® SAT, respectively. In comparison, the estimated mean rate for blunt force trauma was 0.98 [0.948–0.998, 95% CI]. Notwithstanding that increasing the sample size for each device would have resulted in a narrower credible interval, it is unlikely that the rank of the devices would change, so the Blitz Kerner would remain the most effective stunning device among those tested.

## Discussion

This study used behavioural assessment of brain function, changes in EEG and degree of macroscopic damage to the brain and skull to determine the effectiveness of four commercially available CBDs when used on kangaroo pouch young. Stunning produced states of brain activity that were inconsistent with consciousness in 100% (n = 20) of animals that were shot with the Blitz Kerner penetrating captive bolt. Animals shot with this device did not show any behavioural indicators of consciousness after shooting and there was no normal-like active EEG (or non-complete concussion) detected. The majority of animals shot with the Blitz Kerner had moderate to severe macroscopic damage to the thalamus, midbrain, pons, cerebellum and the frontal, parietal and occipital lobes of the cerebrum. However, there was only mild or no macroscopic damage to the medulla in 85% and to the temporal lobe of the cerebrum in 95% of animals.

In the study, seven out of the 29 joeys shot with a non-penetrating CBD had periods of, or recovered, rhythmic breathing but this was not always associated with other behavioural or EEG signs of ineffective stunning. What was common with all of these joeys was either the absence of, or only mild, damage to the medulla which is located in the brainstem. Thus, the damage to the cerebral cortex and other regions of the reticular activating system (thalamus, midbrain and pons) was adequate to induce unconsciousness but insufficient damage to the medulla in the brainstem may have been a factor in preventing the return of rhythmic breathing. This is because the medullary respiratory rhythm generators are in the medulla and are involved in the automated control of respiratory function (inspiratory and expiratory activity) (Ikeda *et al.* 2017). Interestingly, there were other animals (e.g. 35% of those shot with the Blitz Kerner) that also had no macroscopic damage to the medulla but were successfully stunned and did not return to rhythmic breathing. However, it is possible that they may have had microscopic damage to this essential structure (Al-Sarraj 2016). Rhythmic breathing in isolation is not a sign of consciousness, however, its presence in the absence of exsanguination allows oxygenated blood to be delivered to the brain that may support recovery of/maintenance of brain function that could support consciousness. For this reason, it is essential that when functional rhythmic breathing is detected (as opposed to non-functional agonal gasping) even in the absence of other behavioural signs, a second shot is immediately taken. Similarly, this highlights that, even when using a penetrating CBD and stunning is successful with no signs of incomplete concussion, a secondary procedure (such as exsanguination) must still be performed to ensure there is no potential for recovery.

After successful stunning, animals are rendered instantaneously unconscious preventing them from experiencing pain and/or distress. In the EEG this is reflected by a pattern of waveform types before eventually becoming isoelectric, which represents a non-recoverable state of dysfunction (brain death). We observed that the EEG waveforms of successfully stunned joeys generally followed a pattern of transitional or HALF (high amplitude low frequency) activity before becoming isoelectric. Transitional EEG is different from both pre-treatment active and isoelectric EEG and has been characterised as being incompatible with consciousness (or sensibility) in mammals (Gibson *et al.* 2009a,b, 2019; Dalla Costa *et al.* 2020) and birds (Gibson *et al.* 2018). The HALF EEG activity seen in joeys with this study was associated with raised delta and theta power. Previously these EEG states have been reported in cattle (*Bos taurus*) and calves successfully shot with a penetrating captive bolt (Lambooy & Spanjaard 1981; Daly *et al.* 1988; Zulkifli *et al.* 2013; Gibson *et al.* 2019). HALF activity has also been reported in dogs (*Canis familiaris*) after shooting with a penetrating captive bolt (Dennis *et al.* 1988), in humans after traumatic brain injury (Galovic *et al.* 2018) and is seen during unconsciousness caused by concussive impacts (Shaw 2002).

Although we observed periods of normal-like EEG in three of the animals shot with a non-penetrating CBD and in one stunned with manual blunt force trauma to the head, this does not mean that these animals were conscious or could feel pain. A period of normal-like EEG following head trauma has been reported previously in goats (*Capra hircus*) (Sutherland *et al.* 2016) and cattle (Gibson *et al.* 2009a,b) prior to irrecoverable insensibility when shot with non-penetrating captive bolt. So, there is the potential that these animals may be insensible and unable to experience pain and distress, however, these findings could equally indicate a short period of full or reduced consciousness (e.g. shallow depth of concussion) where there is potential for significant welfare compromise. Unlike with the non-penetrating devices, there were no cases of EEG states that could support consciousness in joeys shot with the penetrating CBD. It is possible that the softness and unfused sutures typical of the skulls of joeys could potentially reduce the effectiveness of non-penetrating CBDs. Compared to placental mammals, marsupials have a longer period of brain development and ossification of the skull is much slower (Smith 2006). Fusion of cranial sutures is also delayed and the pattern of fusion of marsupial skulls is different to that of placental mammals (White *et al.* 2025). Skull sutures are functionally important especially to support feeding and accommodating brain growth, but they also act as shock absorbers to absorb strain from other external inputs, such as fighting behaviours and locomotion (White *et al.* 2021). In contrast to placental neonates, in which all or most cranial bones are at least partially ossified prior to suckling, the complete ossification of the skull and fusion of cranial sutures is not complete in marsupials until well into adulthood (Bennett & Goswami 2013). Since non-penetrating CBDs cause concussion from the bolt impacting a hard skull (Finnie 1995), if the skull is soft and pliable and still has unfused cranial sutures some of the forces could be absorbed, rendering these devices less effective at achieving unconsciousness. Thus, although they can be successfully used to euthanase neonates of other species, e.g. goats (Sutherland *et al.* 2016), lambs (Grist *et al.* 2018a), and piglets (Grist *et al.* 2018b), non-penetrating CBDs should not be used on kangaroo joeys as sufficient energy may not reach the brain to cause initial and/or enduring unconsciousness. With penetrating CBDs, previous studies suggest that unconsciousness is caused by a combination of kinetic energy delivered to the animal’s head and direct damage to specific structures of the brain (Gibson *et al.* 2015c). Although the penetrating CBD we tested consistently induced immediate unconsciousness and death without recovery; when using these devices we recommend that field operators follow a precautionary principle approach and apply a secondary killing procedure such as exsanguination and/or decapitation after initial successful stunning to ensure non-recovery and rapid death in every case.

The currently used method for euthanasing pouch young, manual blunt force trauma (or concussive blow) to the head was also evaluated in this study and found to be effective in inducing extensive brain damage resulting in unconsciousness. One of the six joeys killed using this method required two blows as the first did not contact the correct location on the head. The second blow was applied immediately after the first and it is uncertain if the initial blow rendered the animal unconscious and therefore insensible to pain. This is because joeys could not be wired to record EEG whilst the manual blunt force trauma was applied, and behavioural signs of consciousness could not be assessed due to the rapid application of the second blow.

In conclusion, the penetrating CBD tested was effective in rendering 100% of pouch young unconscious while the three non-penetrating devices were effective in only 76% of animals tested. Based on our findings, the Blitz Kerner penetrating captive bolt (used with green activators) is an effective device to achieve unconsciousness in partially to fully furred pouch young during kangaroo harvesting, when it is used in the correct shot position (on the top or crown of the head at the midline aiming slightly back towards the base of the jaw). Immediately after successful stunning, animals must still be exsanguinated (or bled out) to ensure death without recovery.

Factors such as type of device, kinetic energy, shot placement, bolt penetration depth, skull characteristics and operator experience can all potentially influence the effectiveness of CBDs at inducing unconsciousness and the type and extent of damage to brain structures. This study highlights the importance of evaluating different captive-bolt devices on the species and age group upon which they are intended to be used and also demonstrates the advantages of assessing consciousness, unconsciousness and brain death using a range of approaches during the evaluation of a new stunning method. Using only behavioural indicators of consciousness, EEG or brain/skull pathology in isolation might not always provide an accurate or complete picture of the state of the animal or damage caused by the stun.

### Animal welfare implications

The immediacy of action and reproducibility of captive-bolt stunning will improve the welfare of kangaroo pouch young killed during commercial harvesting (and when euthanasia in a field situation is required for other reasons). Although the currently used method of manual blunt force trauma to the head is sufficiently effective in causing extensive brain damage that results in irreversible unconsciousness, different levels of operator skill and confidence in its application could result in variable results. Wherever possible, the use of a penetrating captive-bolt device followed by exsanguination is therefore recommended as the main method for the humane killing of kangaroo pouch young during harvesting. To ensure captive-bolt devices are used correctly, humanely and safely, it is essential that kangaroo shooters receive suitable training in their application, maintenance and storage.

## Supporting information

10.1017/awf.2026.10070.sm001 Sharp et al. supplementary material Sharp et al. supplementary material

## Data Availability

The data that support the findings of this study are available from TS, upon reasonable request.

## Supplementary material

The supplementary material for this article can be found at http://doi.org/10.1017/awf.2026.10070.
