# Supplementary material for: Behavioural and electroencephalographic assessment of captive-bolt stunning in kangaroo pouch young
Source: Anim Welf. 2026 Mar 26;35:e20. doi: 10.1017/awf.2026.10070 (PMC13126202; doi:10.1017/awf.2026.10070)
Supplement: Sharp et al. supplementary material [file S0962728626100700sup001.pdf]

Behavioural and electroencephalographic assessment of captive-bolt stunning in kangaroo pouch young: Supplementary material

Table S1. Performance characteristics of potentially suitable captive-bolt devices.

	**Captive Bolt Model**						
	**TED**	**CASH® SAT (or CPK)**	**CASH® Special**	**Zephyr EXL**	**Blitz Schlag**	**Kleiner Blitz**	**Blitz Kerner**
**Manufacturer**	Bock Industries	Accles & Shelvoke	Accles & Shelvoke	Bock Industries	turbocut Jopp GmbH	turbocut Jopp GmbH	turbocut Jopp GmbH
**Type**	Non-penetrating	Non-penetrating	Penetrating	Non-penetrating	Non-penetrating	Penetrating	Penetrating
**Energy source**	Propane gas	Cartridge	Cartridge	Compressed air	Cartridge	Cartridge	Cartridge
**Powerload**	N/A	1gr brown .22	1gr brown .22	110psi	Green[D] 9x17mm	Green[D] 9x17mm	Green[D] 9x17mm
**Bolt mass (g)**	61	179	211	69	248	209	209
**Mean Peak Velocity (±SD) (m/s)**	30.4 (±0.2)	29.3 (±1.0)	29.8 (±1.2)	26.5 (±0.4)	32.6 (±2.9)	45.3 (±0.5)	47.4 (±0.7)
**Mean Kinetic Energy (±SD) (J)**	28.4 (±0.4)	77.1 (±5.0)	93.9 (±7.5)	24.2 (±0.7)	131.8 (±1.0)	214.3 (±5.2)	234.8 (±7.2)
**Penetration depth or maximum bolt travel distance (±SD) (mm)**	11.7 (with adapter #1)[A]	50[A]	74.0 (±5.1)[B]	27.2[A]	10[A]<br>18.5[C]	52[A]	82[A]<br>65[B]

Notes. Highlighted in blue are the captive bolt devices chosen for pilot testing, the other three are shown for comparison

A=supplied by manufacturer, B=tested in ballistic gel, C=tested in modelling clay, D=Nominal powerload unavailable from manufacturers

Table S2 Cartridge weight loss for Blitz Kerner, Blitz Schlag and CASH® SAT.

CBD	Mean (± SD) cartridge weight loss (g)	Weight loss range (g)	n
**Blitz Kerner**	0.1593 (± 0.0010)	0.15740 - 1616	20
**Blitz Schlag**	0.1579 (± 0.0012)	0.1566 - 0.1604	13
**CASH® SAT**	0.1918 (± 0.0043)	0.1854 - 0.1951	5

Table S3 Macroscopic damage observed in brain structures in the seven red kangaroo (*Osphranter rufus*) pouch young shot with a non-penetrating CBD that had signs of *incomplete* concussion.

Damage to	Severity of damage (%)			
	None (n)	Mild (n)	Moderate (n)	Severe (n)
**Thalamus**	15 (1)	15 (1)	0 (--)	70 (5)
**Midbrain**	15 (1)	15 (1)	30 (2)	40 (3)
**Pons**	15 (1)	70 (5)	15 (1)	0 (--)
**Medulla**	15 (1)	85 (6)	0 (--)	0 (--)
**Cerebellum**	0 (--)	30 (2)	30 (2)	40 (3)
**Frontal lobe**	0 (--)	0 (--)	30 (2)	70 (5)
**Temporal lobe**	55 (4)	15 (1)	30 (2)	0 (--)
**Parietal lobe**	0 (--)	0 (--)	0 (--)	100 (7)
**Occipital lobe**	0 (--)	0 (--)	15 (1)	85 (6)

Table S4. Macroscopic damage observed in brain structures in the 22 red kangaroo (*Osphranter rufus*) pouch young successfully *stunned* with a non-penetrating CBD.

Damage to	Severity of damage (%)			
	None (n)	Mild (n)	Moderate (n)	Severe (n)
**Thalamus**	0 (--)	14 (3)	45 (10)	41 (9)
**Midbrain**	9 (2)	27 (6)	32 (7)	32 (7)
**Pons**	36 (8)	23 (5)	27 (6)	14 (3)
**Medulla**	41 (9)	32 (7)	18 (4)	9 (2)
**Cerebellum**	0 (--)	5 (1)	23 (5)	73 (16)
**Frontal lobe**	0 (--)	9 (2)	18 (4)	73 (16)
**Temporal lobe**	64 (14)	27 (6)	5 (1)	5 (1)
**Parietal lobe**	0 (--)	0 (--)	18 (4)	82 (18)
**Occipital lobe**	0 (--)	0 (--)	14 (3)	86 (19)
